# Are liver metastases from uveal melanoma a clinical indication for dynamic PET? A comparison of Patlak parametric imaging with standard and delayed static SUV imaging using long axial field-of-view PET/CT

**DOI:** 10.1007/s00259-026-08014-x

**Published:** 2026-06-20

**Authors:** Leyun Pan, Christos Sachpekidis, Dimitrios S. Strauss, Jessica C. Hassel, Antonia Dimitrakopoulou-Strauss

**Affiliations:** 1 Clinical Cooperation Unit Nuclear Medicine, German Cancer Research Center (DKFZ), Im Neuenheimer Feld 280, 69210 Heidelberg, Germany; 2 Department of Radiology, University Hospital Heidelberg, Heidelberg, Germany; 3 Heidelberg University, Medical Faculty Heidelberg, Department of Dermatology and National Center for Tumor Diseases (NCT), NCT Heidelberg, a partnership between DKFZ and University Hospital Heidelberg, Heidelberg, Germany

**Keywords:** Long axial field-of-view PET/CT, [^18^F]FDG PET/CT, Patlak parametric imaging, Liver Metastases, Uveal melanoma

## Abstract

**Purpose:**

Despite decades of technical development, dynamic PET and parametric imaging have not yet achieved widespread clinical implementation in oncology, largely due to technical complexity and the limited number of clinical scenarios in which clear additional value over static imaging has been demonstrated. We hypothesized that liver metastases from uveal melanoma—a tumor entity often characterized by low tumor-to-background contrast on static imaging— may represent a particularly promising application for dynamic PET imaging. Accordingly, we aimed to determine whether dynamic Patlak parametric imaging improves lesion detectability and conspicuity compared with standard (50–60 min) and delayed (70–85 min) static standardized uptake value (SUV) imaging.

**Material and methods:**

Twenty-six patients with uveal melanoma and a total of 126 tumor lesions, including 58 liver metastases and 68 other metastases, were prospectively enrolled. All patients underwent a 60 min dynamic data acquisition (skull to upper thigh) followed by whole-body [^18^F]FDG PET/CT imaging at 70 min post-injection. Three image datasets were generated and compared: (1) Direct Patlak K_i_ parametric images derived from dynamic acquisition at 30–60 min post-injection, (2) standard static SUV images reconstructed from same dynamic acquisition at 50–60 min post-injection (standardSUV), and (3) delayed static SUV images acquired at 70–85 min post-injection (lateSUV). All three image sets were assessed qualitatively for lesion detectability and quantitative analysis was performed for all 126 lesions using tumor-to-background ratio (TBR_mean_) measurements.

**Results:**

No significant differences were observed among the three imaging approaches with respect to the total number of detected tumor lesions. However, one discordant finding was noted: a true-positive liver metastasis was identified on Patlak K_i_ imaging and subsequently confirmed by MRI, but was not clearly visualized on static SUV images. Qualitatively, for liver and other metastases, Patlak K_i_ and lateSUV images showed significantly higher TBR_mean_ values than standardSUV, with mean increases of ~ 2.1–2.3-fold for K_i_ and ~ 1.3-fold for lateSUV. Furthermore, Patlak K_i_ yielded significantly higher TBR_mean_ than lateSUV (52/58 and 65/68) and standardSUV (57/58 and 67/68) for liver and other metastases, respectively.

**Conclusions:**

Although lesion detection rates were not significantly increased, dynamic acquisition with Patlak parametric imaging provided a robust and less timing-dependent method to enhance tumor-to-background contrast compared with static SUV imaging and may yield discordant findings with additional clinical value in selected cases. Overall, Patlak imaging may therefore represent a complementary contrast-optimization approach for evaluating liver metastases from uveal melanoma.

**Supplementary Information:**

The online version contains supplementary material available at https://doi.org/10.1007/s00259-026-08014-x.

## Introduction

Dynamic positron emission tomography (PET) and parametric imaging have long been regarded as powerful but underutilized tools in oncologic imaging. While kinetic modeling techniques—such as Patlak graphical analysis—have been available for decades, their translation into routine clinical practice has been limited by technical complexity, prolonged acquisition times, and the fact that static standardized uptake value (SUV) imaging is sufficient for most clinical indications [1–5]. Consequently, the field has continued to search for clinical scenarios in which dynamic PET may provide additional value compared with conventional static imaging.

Liver metastases from uveal melanoma may represent a particularly promising application for dynamic PET imaging. Uveal melanoma is the most common primary intraocular malignancy in adults, and despite effective local control of the primary tumor, up to 50% of patients develop metastatic disease, predominantly in the liver [6–8]. Early and accurate detection of hepatic metastases is critical, as prognosis is poor once systemic dissemination occurs, and therapeutic strategies are increasingly guided by imaging findings [8, 9].

However, PET imaging of liver metastases from uveal melanoma presents specific challenges. Physiologic hepatic uptake of many radiotracers—particularly [^18^F]FDG —may be similar to or only slightly lower than uptake in metastatic lesions during standard imaging windows (typically 50–60 min post-injection) [10]. As a result, tumor-to-background contrast can be limited, reducing lesion conspicuity and sensitivity, particularly for small or infiltrative lesions.

Theoretically, contrast in static PET imaging improves over time as tracer distribution shifts due to metabolism, clearance, or irreversible binding in tumor tissue. Indeed, delayed imaging has been shown in several oncologic settings to enhance lesion detectability [11, 12]. However, the optimal imaging time point is not universal and depends on tracer kinetics, tumor biology, hepatic function, and patient-specific factors. In clinical practice, identifying the ideal static acquisition window is often impractical.

Dynamic PET imaging combined with kinetic modeling offers a fundamentally different approach. Rather than relying on a single time point, dynamic acquisition captures the full time–activity curve, enabling estimation of kinetic parameters such as the net influx rate constant (K_i_) using Patlak analysis for tracers with irreversible trapping [1, 13]. Parametric K_i_ images may enhance tumor detection by suppressing background activity while emphasizing tracer trapping within malignant tissue.

In this study, we investigated whether dynamic [^18^F]FDG PET with Patlak parametric imaging improves the detection and conspicuity of liver metastases from uveal melanoma compared with standard and delayed static imaging. We hypothesized that dynamic parametric imaging can approximate the contrast achievable at the theoretically optimal static time point, thereby providing a robust and less timing-dependent optimization strategy.

## Materials and methods

### Patients

This was a single-center prospective observational study. A total of 26 patients with uveal melanoma and 126 metastases, including 58 liver metastases and 68 other metastases, were prospectively enrolled (mean age 67.3 years; range 40–88 years). Patient characteristics are summarized in Table 1. All patients were scheduled for tebentafusp therapy after this baseline [^18^F]FDG PET/CT for staging. The study was conducted in accordance with the Declaration of Helsinki, and was approved by the Ethics Committee of the University of Heidelberg (S-826/2024). All patients gave written informed consent to undergo [^18^F]FDG and to have their medical records released.

**Table 1:** Characteristics of the patients investigated

Patient Nr	Gen-der	Age	Weight(Kg)	Inj.Dose (MBq)	Metastases	Patient Nr	Gen-der	Age	Weight(Kg)	Inj.Dose (MBq)	Metastases
1	F	65	60	169.87	Liver and Other	14	F	77	59	106.14	Liver
2	M	40	80	158.80	Liver	15	M	79	90	200.13	Liver and Other
3	M	78	85	173.48	Liver	16	F	56	53	111.79	Liver and Other
4	F	68	52	106.16	Liver	17	F	61	57	146.20	Liver
5	F	53	67	134.33	Liver and Other	18	M	62	84	186.35	Liver and Other
6	F	63	44	77.64	Liver and Other	19	M	73	76	150.31	Liver and Other
7	M	88	98	188.59	Liver and Other	20	M	45	121	231.91	Liver
8	M	72	76	160.29	Liver and Other	21	F	72	62	127.63	Liver
9	M	75	61	123.63	Liver	22	M	75	80	169.37	Liver
10	M	68	73	138.29	Liver and Other	23	M	59	93	185.66	Liver and Other
11	F	73	54	108.79	Liver and Other	24	M	71	81	150.24	Liver and Other
12	M	61	78	162.33	Liver and Other	25	M	78	85	173.10	Liver and Other
13	M	65	82	143.58	Liver and Other	26	M	72	106	207.60	Liver and Other

### PET/CT examination

Patients underwent PET/CT with a LAFOV scanner (Biograph Vision Quadra, Siemens Co., Erlangen, Germany) after intravenous administration of a body weight-adjusted activity of 2 MBq/kg [^18^F]-FDG (mean 160 MBq; range 102–270 MBq). Participants fasted for at least 6 h prior to intravenous injection. PET/CT dynamic data acquisition was performed from the top of the head to the upper thigh (FOV 106 cm) for 60 min after i.v. injection of the radiotracer using a 33-frame protocol (10 frames of 15 s, 5 frames of 30 s, 5 frames of 60 s, 5 frames of 120 s, and 8 frames of 300 s). The framing protocol followed the vendor-recommended standard dynamic acquisition workflow used at our institution for dynamic PET studies and parametric imaging on the LAFOV PET/CT system. After the end of the dynamic study, continuous total-body imaging from skull to feet was acquired over 15 min. The whole-body studies of the first seven patients have been acquired with two bed positions, one over the body trunk (10 min) and another over the extremities (5 min). Furthermore, the PET images of these first seven patients were reconstructed using HS mode (18° acceptance angle). This was due to the fact that continuous total-body imaging and ultra-high-resolution mode (UHS) were not available by the manufacturer at the installation time of the LAFOV system in 2021. All other PET images were reconstructed using UHS mode (52° acceptance angle). Images were attenuation-corrected and iteratively reconstructed using the manufacturer’s standard reconstruction method (Siemens Healthineers) using the point spread function + time-of-flight algorithm (PSF + TOF, 4 iterations × 5 subsets) without Gaussian filtering into 1.65 × 1.65 × 1.65 mm^3^ voxels and an image matrix of 440 × 440 pixels. A low-dose attenuation CT (120 kV, 30 eff. mA) was used for attenuation correction of the dynamic emission PET data and for image fusion.

### Patlak imaging

The parametric images (FOV: 106 cm) were generated using the direct Patlak method. Automatic image segmentation of the descending aorta served as the image-derived input function (IDIF). Direct Patlak reconstruction was performed using the manufacturer-provided implementation (Siemens Healthineers). In the first seven patients, parametric reconstruction was performed using e7-tools due to software availability at the time of scanner installation, whereas later datasets were reconstructed using the integrated vendor implementation. Following recommendations from the experts of the manufacturer, we used the last 30 min of the dynamic acquisition protocol in 6 frames (last 6 frames of 300 s) to reconstruct the Patlak K_i_ images. Two datasets are provided by the Patlak reconstruction, the distribution volume (DV) images and K_i_ images. DV images are related to FDG transport whereas K_i_ images are related to phosphorylation. In this evaluation we focused on the K_i_ images.

### Data analysis

#### Visual assessment of Patlak parametric images

Patlak parametric image analysis was performed using a dedicated imaging workstation and software (aycan Osirix^PRO^ and PMOD). Two experienced, board-certified nuclear medicine physicians well versed in oncologic PET and pharmacokinetic modeling (CS, ADS) reviewed the datasets together, and any disagreements were resolved by consensus.

Visual analysis was based on the identification of sites of focally enhanced [^18^F]FDG uptake relative to liver background, which were considered suggestive of tumor involvement (liver metastases or other metastases) after excluding known benign [^18^F]FDG avid structures, such as sites of unspecific uptake after comparison with low dose CT and patient history. The number of tumor lesions was determined in each scan, with a maximum of up to 20 lesions recorded per patient. Three image datasets were generated and compared: (1) Direct Patlak K_i_ parametric images derived from dynamic acquisition, (2) standard static SUV images acquired at 50–60 min post-injection (standardSUV), and (3) delayed static SUV images acquired at 70–85 min post-injection (lateSUV). Lesion detectability was compared across the three image datasets.

All patients had known metastatic liver disease, based on prior MRI imaging and biopsy diagnostic, and were scheduled for treatment with tebentafusp. Other diagnostic imaging modalities, such as contrast-enhanced CT and MRI served as a reference in selected cases. Metastatic disease was histologically confirmed in particular for liver metastases.

#### Comparing different images sets from the dynamic scan and static scan

Parametric images and standard SUV images were reconstructed from the same dynamic PET acquisition. Therefore, VOIs could be directly transferred between Patlak Ki images and standard SUV images, allowing direct comparison between the two datasets.

Delayed static images (lateSUV) were obtained in a separate acquisition performed after completion of the dynamic scan. Between the two acquisitions, patients were allowed to leave the scanner table if necessary (e.g., for bladder voiding) and were subsequently repositioned for the delayed scan. Consequently, lateSUV images represent a different study, rendering direct comparison with the images reconstructed from the dynamic dataset challenging. Manually delineating VOIs independently on both studies would be time-consuming and could introduce inconsistencies in VOI definition. To address this issue, we first performed image registration between the two studies using PMOD software and saved the resulting transformation matrix. VOIs were initially defined on the delayed static images (lateSUV). These VOIs were then transferred to the images reconstructed from the dynamic acquisition by applying the saved transformation matrix. All transferred VOIs were subsequently reviewed by reading physicians (CS, ADS), who manually corrected—if needed—minor discrepancies resulting from the automatic registration process. This workflow enabled efficient and consistent VOI definition across both the dynamic and static datasets, thereby allowing direct and reliable comparison of Patlak K_i_ images, standard SUV images, and lateSUV images.

#### Objective evaluation of Patlak parametric image quality

Evaluation of the dynamic PET/CT data was performed based on VOIs drawn over liver metastases as well as other metastases and normal, non-affected liver tissue for reference. In particular, liver metastases were assessed using irregular VOIs drawn with an isocontour mode encompassing the entire lesion. For normal liver tissue, spherical VOIs covering approximately five consecutive slices were placed using an isocontour mode. Blood pool activity was calculated from the descending aorta using VOIs consisting of at least seven slices in sequential PET/CT images, placed centrally within the lumen while avoiding inclusion of the aortic wall.

To quantitatively compare static SUV images and K_i_ images, target-to-background ratios (TBR) for individual liver metastases and other metastases were calculated as described in Eqs. 1, using liver parenchyma as background:

1 $${\mathrm{T}\mathrm{B}\mathrm{R}}_{mean} =\frac{\mathrm{M}\mathrm{e}\mathrm{a}\mathrm{n}\left({\mathrm{V}\mathrm{O}\mathrm{I}}_{\mathrm{L}\mathrm{e}\mathrm{s}\mathrm{i}\mathrm{o}\mathrm{n}}\right)}{\mathrm{M}\mathrm{e}\mathrm{a}\mathrm{n}\left({\mathrm{V}\mathrm{O}\mathrm{I}}_{\mathrm{B}\mathrm{a}\mathrm{c}\mathrm{k}\mathrm{g}\mathrm{r}\mathrm{o}\mathrm{u}\mathrm{n}\mathrm{d}}\right)}$$

### Statistical analysis

Image quality parameter (TBR_mean_) for Patlak Ki, standardSUV, and lateSUV images was summarized as mean, standard deviation (SD), minimum, and maximum. Differences among the three groups were assessed using the Wilcoxon matched-pairs signed-rank test. Statistical analysis was performed with Stata/MP 14.2 (StataCorp LLC).

## Results

Twenty-six patients with uveal melanoma and a total of 126 metastases were evaluated. Direct Patlak K_i_ parametric images derived from dynamic acquisition were feasible and of good image quality. We compared all three image datasets, namely direct Patlak K_i_ parametric images derived from dynamic acquisition, standard static SUV images acquired at 50–60 min post-injection (standardSUV), and delayed static SUV images acquired at 70–85 min post-injection (lateSUV). All three image sets were assessed qualitatively for lesion detectability, and quantitative analysis was performed for all 126 lesions using TBR measurements.

**Fig. 1:**
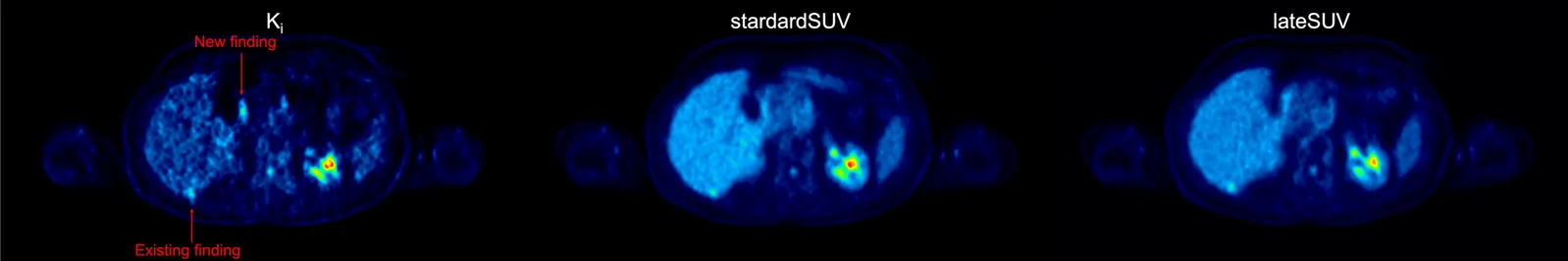
In patient No. 21, besides a small liver metastasis delineated in segment VII, an additional lesion in segment IV was identified on Patlak K_i_ imaging(arrow) and subsequently confirmed as a liver metastasis on MRI, but was not clearly visualized on static SUV images

**Fig. 2:**
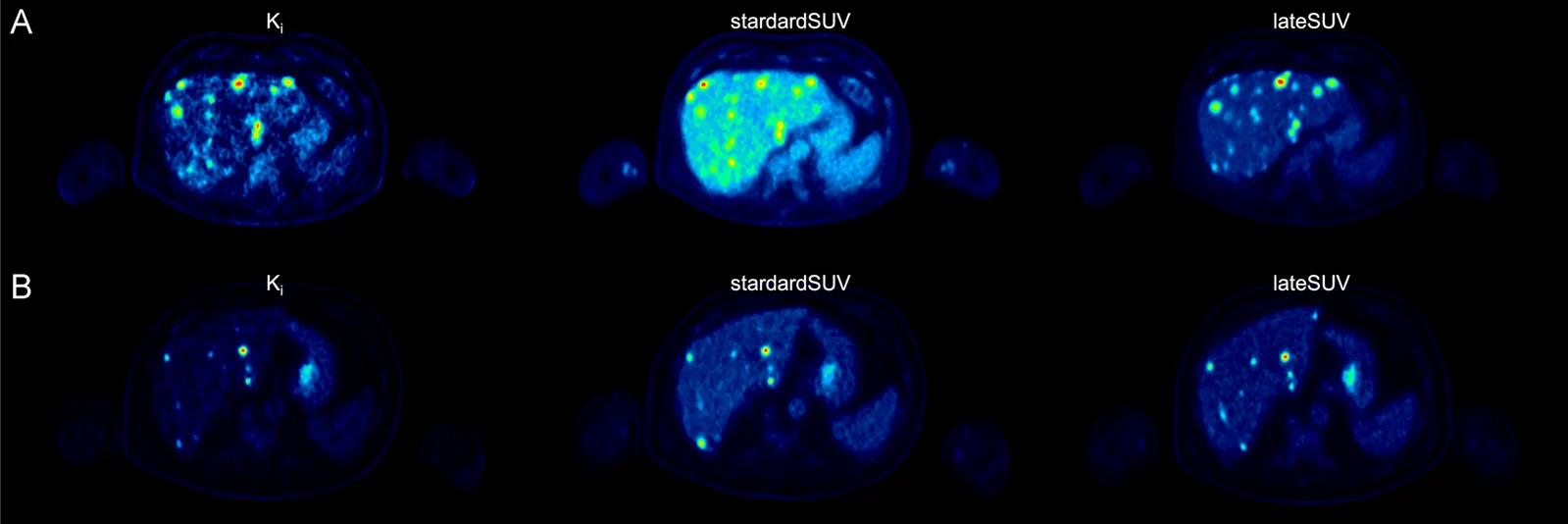
In patient No. 22 (row A), lesion conspicuity was greater on late SUV images compared with K_i_ images, with moreover, more lesions detected and clearly improved delineation relative to standard SUV images. In patient No. 7 (row B), a higher number of lesions was detected on late SUV images compared with both K_i_ Patlak and standard SUV images

**Fig. 3:**
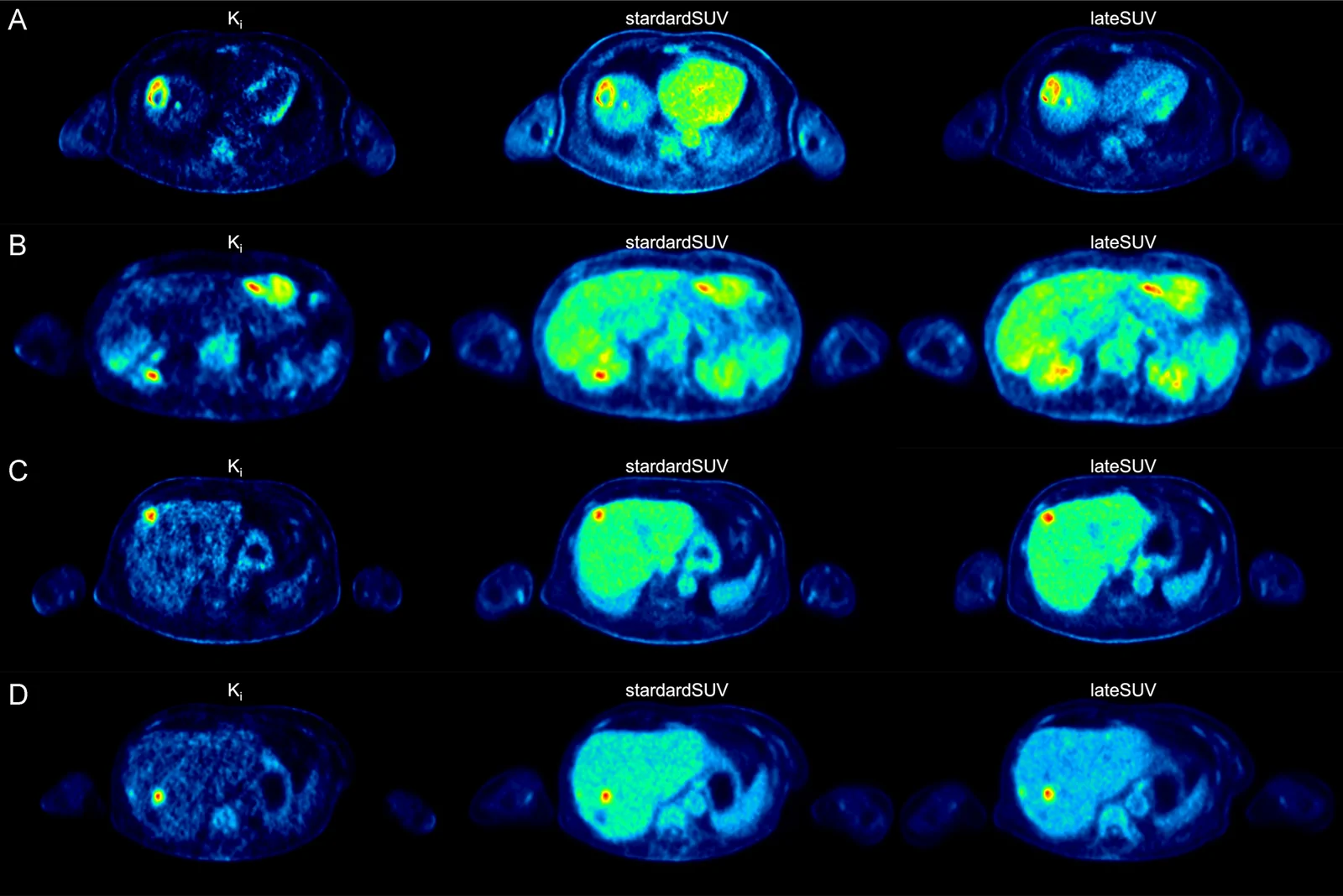
In patients No. 3, 6, 10, and 14 (rows A–D), Patlak K_i_ images demonstrated markedly higher lesion contrast for liver metastases compared with static images, whereas late SUV static images provided superior contrast compared to standard SUV static images

No significant differences were observed among the three imaging approaches with respect to the total number of detected tumor lesions. However, we noted one discordant finding: a true-positive liver metastasis was identified on Patlak K_i_ imaging and subsequently confirmed by MRI, but was not clearly visualized on static SUV images, both standard and late (Fig. 1). In two additional cases, better delineation was observed on lateSUV images compared with K_i_ images (Fig. 2), indicating that K_i_ imaging is not uniformly superior to delayed static imaging. Qualitatively, most K_i_ images demonstrated very high lesion contrast for liver metastases (Fig. 3), while lateSUV images generally showed improved contrast compared with standardSUV images.

**Fig. 4:**
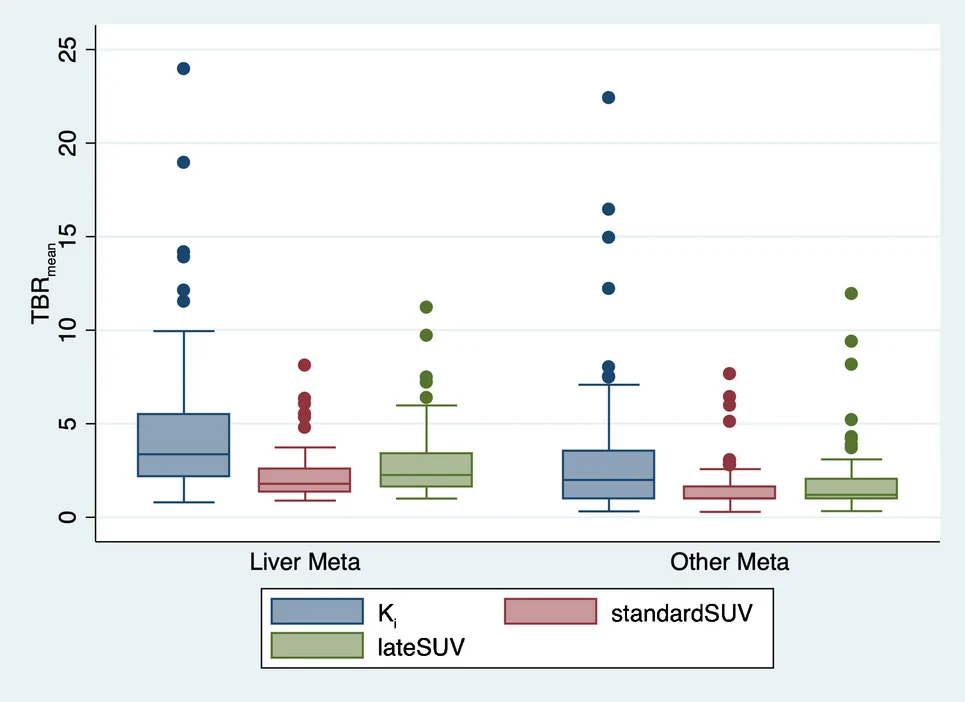
Boxplots of TBR_mean_ for Patlak K_i_, standardSUV and lateSUV grouped by liver metastases and other metastases

TBR_mean_ values for 58 liver metastases and 68 other metastases (Table 2) were consistent with the overall findings. For both liver and other metastases, Patlak K_i_ and lateSUV images showed significantly higher TBR_mean_ values than standardSUV, with mean increases of ~ 2.1–2.3-fold for K_i_ and ~ 1.3-fold for lateSUV. Boxplots of TBR_mean_ for the three imaging methods, stratified by metastases type, are shown in Fig. 4. Furthermore, Wilcoxon matched-pairs signed-rank test results (Table 3) demonstrated that Patlak K_i_ yielded significantly higher TBR_mean_ than lateSUV (52/58 and 65/68) and standardSUV (57/58 and 67/68) for liver and other metastases, respectively.

**Table 2:** Descriptive statistics of TBR_mean_ for Patlak Ki, standard SUV, and late SUV in liver metastases and other metastatic lesions, using normal liver parenchyma as the reference background

liver metastases/other metastases	Obs	Mean	SD	Min	Max
TBR_mean_ of K_i_	58/68	4.91/3.85	4.40/3.82	0.80/0.32	23.97/22.37
TBR_mean_ of standardSUV	58/68	2.31/1.69	1.47/1.36	0.89/0.29	8.12/7.63
TBR_mean_ of lateSUV	58/68	2.98/2.15	2.08/1.94	1.09/0.33	11.20/11.90

**Table 3:** The number of positive ranks from the Wilcoxon signed-rank test for three TBR_mean_ datasets, stratified by liver metastases (*n* = 58) and other metastatic lesions (*n* = 68), is presented. All pairwise comparisons showed statistically significant differences

liver metastases/other metastases	K_i_	lateSUV	standardSUV
Ki	0/0	52 in 58*/65 in 68*	57 in 58*/67 in 68*
lateSUV		0/0	49 in 58*/61 in 68*
standardSUV			0/0

^*^ Row label is significantly higher than the column label (*p* = 0.0000)

## Discussion

Uveal melanoma is a rare malignancy but represents the most common primary intraocular tumor in adults [14]. It accounts for approximately 3%−5% of all melanomas, with most tumors originating from the choroid [15, 16]. Although both uveal and cutaneous melanomas originate from melanocytes, their pathogenesis and clinical behavior differ substantially and are generally more complex in uveal melanoma [17]. Approximately half of patients with uveal melanoma develop distant metastases, with the liver being the most predominate site [18]. The presence of distant metastases is associated with poor prognosis [9, 19]. Notably, half of the patients with liver metastases exhibit also extrahepatic involvement, mainly in the lungs (30%), bone (23%) and skin (17%) [20].

The treatment of metastatic uveal melanoma remains challenging, as these tumors show limited response to immune checkpoint inhibitors (ICI), such as anti-CTLA-4 and anti-PD-1 antibodies, which are effective in cutaneous melanoma. Outcomes have improved with the introduction of tebentafusp, a first-in-class T-cell receptor–bispecific fusion protein targeting glycoprotein 100 (gp100) and CD3, which redirects T cells to kill gp100-positive melanoma cells. It is currently the only approved systemic therapy for adult HLA-A*02:01–positive patients with unresectable or metastatic uveal melanoma [21] with a survival benefit as shown in a phase 3 study by Hassel et al. In this study the authors reported a median overall survival of 21.6 months in the tebentafusp group versus 16.9 months in the control group (hazard ratio for death, 0.68; 95% confidence interval, 0.54 to 0.87). The estimated percentage of patients surviving at 3 years was 27% in the tebentafusp group and 18% in the control group [22].

A major challenge often observed in imaging liver metastases of uveal melanoma is limited lesion conspicuity due to relatively low [^18^F]FDG uptake compared with the physiologic liver background uptake. This issue is particularly relevant for small hepatic lesions or diffuse infiltration of the liver parenchyma. One strategy to improve lesion delineation is to enhancing TBR, either through late imaging or through dynamic imaging with parametric reconstruction. In the present study, we demonstrate that dynamic [^18^F]FDG PET imaging with Patlak parametric reconstruction improves lesion conspicuity in liver metastases compared with conventional static SUV imaging. While delayed static imaging (70–85 min post-injection) yielded superior contrast compared with standard imaging (50–60 min), Patlak K_i_ images frequently provided further increases in tumor-to-background contrast. These findings suggest that dynamic PET may help mitigate one of the principal limitations of static imaging, namely its strong dependence on acquisition timing.

### Timing dependency of static PET

Static SUV imaging represents tracer concentration at a single time point and is therefore inherently sensitive to tracer kinetics. In the liver, background uptake may remain relatively stable or decline slowly over time, while tumor uptake may continue to increase due to irreversible trapping mechanisms. This temporal divergence can improve contrast in delayed imaging, as previously shown in multiple tumor types [11, 12]. Supplementary Figure S1 illustrates the mean TAC behavior of blood pool activity, normal liver tissue, and liver metastases during dynamic acquisition.

Our findings confirm that delayed imaging improves lesion conspicuity in hepatic metastases from uveal melanoma. However, the magnitude of improvement was variable across patients, likely reflecting interindividual differences in hepatic metabolism, tumor perfusion, expression of relevant transporters or enzymes, and systemic tracer clearance. These factors make it difficult to define a universally optimal static acquisition window. We acknowledge that the standardSUV window used in the present study (50–60 min post-injection) is slightly earlier than the 60-min acquisition time commonly used in clinical routine, and that the delayed imaging window (70–85 min post-injection) does not represent very late imaging. Nevertheless, the comparison between different imaging windows remains informative regarding the timing dependency of lesion conspicuity and tumor-to-background contrast.

### Mechanistic advantages of parametric imaging

Patlak analysis, first described in 1983 [13], enables estimation of the net influx rate constant (K_i_) under conditions of irreversible tracer trapping. Unlike SUV, which reflects a composite of delivery, distribution, and clearance at a single time point, K_i_ isolates the irreversible trapping component of tracer kinetics. This results in suppression of background liver activity, which often reflects reversible tracer distribution, and amplification of tumor signal driven by irreversible metabolic trapping. This phenomenon is also consistent with the known hepatic dephosphorylation of FDG-6P, which contributes to relatively low liver background signal on K_i_ images while liver activity remains more prominent on DV-related images. This dual mechanism enhances tumor-to-background contrast beyond what can be achieved by simple delayed imaging. Importantly, parametric images are less sensitive to the exact acquisition timing, provided that the dynamic acquisition covers the appropriate post-injection window and the assumptions of the kinetic model are met.

Our results align with previous studies demonstrating the added value of parametric imaging in oncologic PET, including improved lesion conspicuity and reduced variability compared with SUV-based assessment [2, 23–25]. Importantly, the feasibility of parametric Patlak imaging is not limited to LAFOV PET systems. Previous studies have also demonstrated clinically feasible multiparametric PET imaging protocols on SAFOV PET systems, including approaches using population-based input functions and shortened dynamic acquisition protocols [26]. In addition, clinically feasible shortened dynamic acquisition protocols have been proposed even for SAFOV PET systems using population-based input functions, enabling multiparametric Patlak imaging within approximately 15–20 min [27]. Such approaches may further facilitate broader clinical implementation of dynamic parametric imaging.

In a recent work [28], we compared direct and indirect Patlak imaging in a cohort of 50 oncological patients with 346 tumor lesions, using different time frames for parametric image reconstruction. All these PET-CT studies have been performed with the same LAFOV system as in the present study. In particular, four sets of images were generated based on the last 30 min of dynamic acquisition as well as using nearly the full acquisition duration (59.25 min), applied to both direct and indirect Patlak parametric methods. As in the current analysis, no significant differences were observed among the four approaches regarding lesion detectability. However, three discordant findings were identified: a true positive liver lesion in all Patlak K_i_ images, a false positive liver lesion delineated only in late-time indirect (LTI) K_i_, subsequently characterized as hemangioma on MRI, and a true negative case involving an atelectasis adjacent to a lung tumor. Notably, K_i_ images demonstrated higher TBR in comparison with static SUV images, in agreement with the present study. In the present study we noted one discordant finding, namely a true-positive liver metastasis delineated on Patlak K_i_ imaging and subsequently confirmed by MRI, but not clearly visualized on static SUV images (Fig. 1).

An important observation is that late static imaging can, in some cases, outperform parametric imaging. In our cohort, two liver metastases were better delineated on lateSUV images than on K_i_ images (Fig. 2), indicating that parametric imaging is not universally superior. Overall, we could demonstrate that lateSUV static images demonstrated better contrast than standardSUV static images. Furthermore, both Patlak K_i_ and lateSUV imaging significantly improved contrast compared with standard SUV imaging, with mean TBR increases of 2.3-fold and 1.3-fold, respectively (Fig. 4). These observations further support the clinical value of delayed static imaging for contrast optimization in hepatic metastases from uveal melanoma and suggest that Patlak imaging and delayed static imaging should be regarded as complementary approaches rather than universally superior alternatives. Although delayed whole-body imaging was acquired using continuous bed motion, we did not observe a relevant reduction in image quality or lesion conspicuity. This may be related to the long acquisition duration and the high sensitivity of the LAFOV PET/CT system.

### Clinical implications

From a clinical perspective, the key question is whether dynamic PET provides actionable benefits over optimized static imaging. In many oncologic scenarios, static PET is sufficient for diagnosis and staging. However, liver metastases from uveal melanoma represent a particularly challenging setting due to high physiological hepatic background uptake, and the prognostic importance of early detection. In this context, even modest improvements in contrast may translate into meaningful diagnostic gains. If dynamic acquisition over a standardized time window (e.g., 30–60 min post-injection) can consistently produce high-contrast parametric images comparable to or superior to the ideal delayed static image, it may eliminate the need for individualized optimization of scan timing. We acknowledge that the different image sets in the present study were reconstructed from acquisition windows of different durations; however, the primary aim was to compare clinically relevant imaging workflows rather than to determine the theoretically optimal static SUV acquisition window. Thus, rather than viewing dynamic PET solely as a niche research tool, it may also be considered a promising contrast-optimization strategy, particularly in organs with high physiologic background uptake such as the liver.

## Limitations

Several limitations merit consideration. First, dynamic imaging requires longer acquisition times and dedicated, more complex reconstruction workflows, which may limit patient throughput in clinical practice. Second, Patlak analysis relies on assumptions of irreversible tracer trapping and accurate input function estimation; deviations from these assumptions may introduce bias [1, 13]. Third, the Patlak method depends on the time-to-equilibrium (t*), which ideally should be fitted to the individual time–activity curves (TACs), but was fixed at 30 min post-injection in the present study [28, 29]. Although shorter early-frame durations may improve characterization of the bolus phase of the input function, Patlak analysis primarily depends on the late linear phase and integrated tracer kinetics. Furthermore, although Patlak imaging is generally less dependent on exact acquisition timing once the linear steady-state phase has been reached, residual variability related to tracer kinetics and tissue-specific equilibrium behavior may still influence image contrast and quantitative parameters. In addition, lesion-level analyses may be affected by intra-patient correlation, since multiple lesions originated from the same patients. Future studies with larger cohorts should evaluate clustered or mixed-effects statistical approaches to account for within-patient dependence. Finally, larger prospective studies are needed to quantify sensitivity gains and to assess impact on clinical management and outcomes.

## Future directions

Technological advances, including total-body PET systems, improved motion correction, automated image-derived input functions, and AI-assisted parametric reconstruction, may substantially reduce current practical barriers to dynamic imaging [30, 31]. As acquisition and reconstruction technologies evolve, the incremental cost and complexity associated with dynamic protocols may decrease, facilitating broader clinical adoption.

Future research should prioritize prospective comparisons between dynamic imaging and optimized delayed static protocols, as well as systemic evaluation of diagnostic accuracy and interobserver variability, and reproducibility. In addition, integration of parametric metrics into treatment response assessment and prognostic modeling represents an important area for further investigation.

## Conclusion

Liver metastases from uveal melanoma represent a particularly promising application for dynamic PET and parametric imaging. While delayed static imaging improves lesion contrast compared with standard acquisition times, the optimal timing remains variable across patients. Dynamic acquisition with Patlak reconstruction provides a robust, less timing-dependent approach to enhance tumor-to-background contrast. In this context, dynamic PET may serve as a complementary contrast-optimization strategy in challenging oncologic scenarios characterized by high physiologic background uptake.

## Supplementary Information

Below is the link to the electronic supplementary material.Supplementary file1 (DOCX 213 KB)

## Data Availability

The datasets generated during and/or analysed during the current study are available from the corresponding author on reasonable request.
