# Supplementary material for: Are liver metastases from uveal melanoma a clinical indication for dynamic PET? A comparison of Patlak parametric imaging with standard and delayed static SUV imaging using long axial field-of-view PET/CT
Source: Eur J Nucl Med Mol Imaging. 2026 Jun 20;53(11):6549–58. doi: 10.1007/s00259-026-08014-x (PMC13526073; doi:10.1007/s00259-026-08014-x)
Supplement: Supplementary file 1 [file 259_2026_8014_MOESM1_ESM.docx]

**Supplementary Figure S1.**
Mean time–activity curves (TACs) during dynamic ^[18F]^FDG PET acquisition for blood pool activity, normal liver tissue, liver metastases, and low-uptake liver metastases.

(A) Mean TACs without standard deviation bars for improved visualization of temporal uptake behavior.
(B) Mean TACs shown with standard deviation bars.

For illustrative purposes, low-uptake liver metastases were defined as lesions with SUVmean < 4 (14/58 liver metastases).
